# Progress in the Application of Machine Learning in the Field of Single-Cell and Spatial Transcriptomics

**DOI:** 10.3390/genes17060604

**Published:** 2026-05-26

**Authors:** Yan Zhu, Ziling Hao, Li Zhu, Linyuan Shen, Yihui Liu, Mailin Gan

**Affiliations:** 1 Farm Animal Germplasm Resources and Biotech Breeding Key Laboratory of Sichuan Province, Sichuan Agricultural University, Chengdu 611130, China; 2024302105@stu.sicau.edu.cn (Y.Z.); 2022002001@stu.sicau.edu.cn (Z.H.); zhuli@sicau.edu.cn (L.Z.);; 2 State Key Laboratory of Swine and Poultry Breeding Industry, Sichuan Agricultural University, Chengdu 611130, China; 3 Key Laboratory of Livestock and Poultry Multi-Omics, Ministry of Agriculture and Rural Affairs, College of Animal and Technology, Sichuan Agricultural University, Chengdu 611130, China; 4 Sichuan Province General Station of Animal Husbandry, Chengdu 610066, China

**Keywords:** machine learning, deep learning, transcriptomics, RNA-sequencing, single-cell transcriptomics, spatial transcriptomics, spatiotemporal transcriptome

## Abstract

The rapid evolution of transcriptome sequencing technologies has driven significant breakthroughs across the life sciences. The advent of single-cell RNA-sequencing (scRNA-seq) has enabled gene expression profiling at single-cell resolution, whereas spatial transcriptomics further contextualizes these transcriptional profiles within preserved tissue morphology. Concurrently, advancements in artificial intelligence have introduced unprecedented opportunities in bioinformatics. As a core component of artificial intelligence, machine learning (ML) substantially outperforms traditional computational methods in deciphering complex, high-dimensional biological data. This review systematically summarizes the significant advantages of integrating ML algorithms into transcriptomic workflows. By leveraging these advanced computational tools, researchers can efficiently extract comprehensive biological insights, elucidate intricate Gene Regulatory Networks, and generate intuitive visualizations. Ultimately, ML-driven transcriptomics provides a robust technical foundation for disease diagnosis, drug discovery, and precision medicine. These advancements underscore the pivotal role of ML in transforming transcriptomic data analysis into an intelligent, highly precise, and multidimensional discipline, thereby accelerating future biological discoveries.

## 1. Introduction

Transcription is the process by which RNA is synthesized from a DNA template via complementary base pairing. Broadly defined, the transcriptome encompasses the complete set of RNA transcripts, including messenger RNA (mRNA), ribosomal RNA (rRNA), transfer RNA (tRNA), and non-coding RNA (ncRNA), produced under specific physiological conditions. Transcriptomic analysis facilitates the molecular-level investigation of gene expression dynamics across specific cells, tissues, or organs during various developmental stages or under diverse environmental stimuli.

Initially, the primary bottleneck in transcriptomic studies was the efficient generation of sequencing data. The advent of next-generation sequencing (NGS) [1] has largely overcome this limitation. Consequently, high-throughput transcriptomic profiling is now widely utilized to elucidate gene expression patterns, investigate disease pathogenesis, and characterize crop stress resistance. However, this technological shift has introduced new analytical challenges. The exponential growth of sequencing data yields highly complex, multidimensional expression profiles that vary significantly across cell types, developmental phases, and environmental contexts.

Relying solely on classical univariate statistical tools is increasingly inadequate for deciphering such complex data [2]. Furthermore, advanced modalities like single-cell and spatial transcriptomics are highly computationally intensive and present significant interpretational hurdles [3,4]. As the pace of data generation far outstrips the capacity of manual analysis, vast amounts of transcriptomic data in public repositories remain underutilized. To address these high-dimensional challenges, modern analytical pipelines do not abandon classical methods; rather, they seamlessly integrate foundational statistical techniques (e.g., principal component analysis, PCA) as preliminary dimensionality reduction steps within broader, highly automated machine learning (ML) frameworks. Although early applications of ML to gene expression data emerged in the early 21st century, these initial approaches lacked accessibility and were not incorporated into standardized analytical workflows [5].

**Figure 1:**
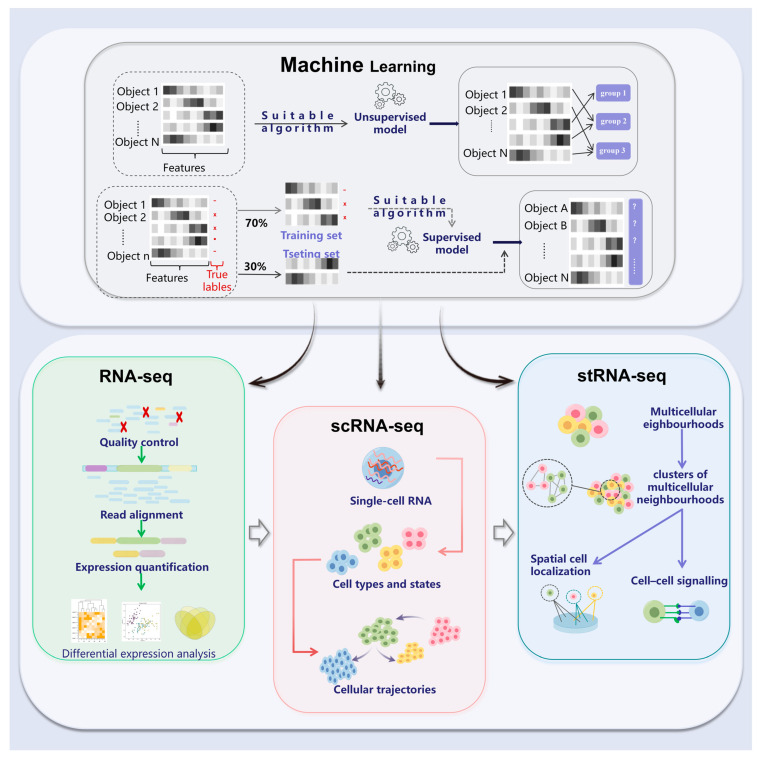
Machine learning has been applied in the analysis flow in various fields of the transcriptome. Machine learning algorithms are mainly divided into supervised and unsupervised categories. Machine learning techniques with these two algorithms as the core have been widely used in various fields of transcriptomics.

In recent years, artificial intelligence (AI) has experienced unprecedented growth, driven largely by significant advancements in machine learning (ML) and the rapid emergence of deep learning technologies [6,7]. Consequently, ML has been extensively integrated into biological and medical research, extending even into routine clinical practice [8,9,10,11,12]. The application of ML algorithms to transcriptomic analysis has become a standard paradigm; for instance, methods such as support vector machines (SVMs) and clustering are now routinely employed for differential expression and enrichment analyses [13,14] (Figure 1). As robust computational tools, ML models efficiently extract critical biological features from massive datasets, thereby accelerating research in animal genetics and breeding, disease diagnosis, epigenetic regulation, and beyond.

Before discussing specific applications, it is essential to define “machine learning” clearly within the context of transcriptomics and to delineate its relationship with classical statistics. Historically, classical statistical methods, such as linear or logistic regression, linear discriminant analysis (LDA), and regularized models (e.g., Lasso and Elastic Net), were firmly rooted in statistical inference, focusing on parameter estimation and hypothesis testing under strict distributional assumptions. However, in the modern omics era, the boundary between statistics and machine learning has evolved into a continuum. When repurposed to optimize predictive accuracy and perform automated feature selection on high-dimensional, highly collinear transcriptomic data, these classical methods function as foundational “statistical learning” algorithms under the broader ML umbrella [15]. Modern deep learning extends this continuum by deploying complex, multi-layered neural networks that bypass rigid statistical assumptions to capture highly non-linear biological representations.

Given the exponential growth of literature in this field, an exhaustive catalog of ML applications across single-cell and spatial transcriptomics is unfeasible. Therefore, this review is intentionally curated to establish a cohesive evolutionary narrative [3]. Rather than treating advanced methodologies as isolated tools, it is crucial to understand their relationships as a continuous computational evolution driven by increasingly complex biological bottlenecks [4].

We trace this progression by examining how the field iteratively evolved to resolve the core challenges of data representation, normalization, and integration. Initially, classical statistical learning methods (such as PCA and linear regression) were sufficient for bulk RNA-seq, providing essential dimensionality reduction for continuous, densely populated matrices. However, as the field transitioned to single-cell resolution, the extreme sparsity (dropout events) and massive batch effects inherent in scRNA-seq rendered rigid linear assumptions inadequate [16]. This biological bottleneck necessitated a leap toward deep generative models (such as VAEs). Architectures like scVI [17] fundamentally revolutionized normalization and integration by projecting cells into non-linear, probabilistic latent spaces, inherently impute missing values and seamlessly correcting batch effects without strict parametric constraints.

Yet, deep generative models still process cells as isolated transcriptomic bags, ignoring the critical biological context of the tissue microenvironment. The subsequent advent of spatial transcriptomics required a methodology capable of interpreting physical topology [18]. Consequently, Graph Neural Networks (GNNs) were adopted to explicitly bridge this gap, modeling spatial coordinates and cell–cell communication networks by treating physical proximity as mathematical edges [19,20]. Finally, while GNNs excel at localized spatial tasks, they typically require retraining for new datasets and struggle with cross-tissue generalizations. To resolve this ultimate challenge of universal representation, the field is currently adopting Transformer-based foundation models [21]. Pre-trained on massive, multi-tissue corpora, these architectures utilize self-attention to capture global gene–gene dependencies, enabling zero-shot predictions and universal cell embeddings.

By connecting these distinct mathematical innovations through the lens of algorithmic evolution, this review systematically illustrates how each architectural paradigm directly compensated for the biological and computational limitations of its predecessor.

## 2. Modern Machine Learning Architectures in Transcriptomics

As established in the preceding historical trajectory, the application of machine learning (ML) to transcriptomics has transitioned from isolated algorithmic sorting into a deeply integrated computational continuum. Historically, ML applications in biology were broadly dichotomized into supervised and unsupervised learning tasks [5]. At its foundational core, deep learning (DL) transcended these rigid boundaries by deploying artificial neural networks with multiple hidden layers to automatically extract hierarchical feature representations directly from raw expression matrices. In contrast to classical ML methods, which typically rely on shallow architectures and manual feature engineering, DL inherently captures complex, non-linear biological relationships. This capacity for automated representation learning makes it exceptionally suited for analyzing high-dimensional transcriptomic profiles.

While these fundamental statistical and algorithmic categorizations remain conceptually relevant, the exponential growth of transcriptomic data—shifting from dense bulk matrices to sparse single-cell profiles and intricate spatial topologies—has driven the field toward highly specialized, task-specific DL architectures [16]. Consequently, modern analytical pipelines have evolved far beyond basic, dense neural networks. To provide a rigorous, problem-oriented analysis of current methodologies, this section classifies modern computational frameworks not as a fragmented list of software packages but according to their underlying architectural paradigms, each designed to overcome a distinct biological bottleneck: deep generative models for sparsity and dropout correction, graph-based representation learning for microenvironmental topology mapping, attention mechanisms for global gene regulatory modeling, and the modern adaptation of classical algorithms for highly interpretable downstream validation.

### 2.1. Deep Generative Models and Latent Space Learning

Single-cell RNA-sequencing (scRNA-seq) datasets are inherently noisy and highly sparse, presenting significant computational challenges such as severe batch effects and “dropout” events—instances where expressed transcripts fail to be detected [16]. Traditional linear dimensionality reduction techniques frequently fail to delineate the complex, non-linear manifolds characteristic of such biological data. Consequently, deep generative models, particularly Autoencoders (AEs) and Variational Autoencoders (VAEs), have emerged as foundational computational architectures.

These models operate by compressing high-dimensional gene expression profiles into a lower-dimensional, dense latent space before reconstructing the original data. This information bottleneck architecture forces the network to learn essential, denoised biological variations while effectively discarding technical noise. In modern analytical pipelines, VAEs function as standard instruments for missing value imputation, batch effect correction, and multi-omic data integration. For instance, the widely adopted scvi-tools framework leverages probabilistic VAEs to facilitate scalable, end-to-end single-cell analyses [22]. Furthermore, architectural innovations such as scArches integrate deep generative models with transfer learning to project novel single-cell query datasets onto massive reference atlases without the computationally prohibitive need to retrain models from scratch. This approach efficiently mitigates batch effects and enhances the identification of rare cell states [23].

### 2.2. Graph-Based Representation Learning

Cells do not operate in isolation; their developmental trajectories, signaling interactions, and spatial localizations are fundamentally interconnected. Graph Neural Networks (GNNs) and graph convolutional networks (GCNs) have revolutionized the field by enabling computational models to explicitly capture and represent these complex, higher-order structural relationships.

Within a graph-based framework, individual cells or spatial capture spots are conceptualized as nodes, while their physical proximities or signaling interactions are defined as edges. This architecture is indispensable for spatial transcriptomics, where spatial context is critical for decoding the tissue microenvironment. Pioneering frameworks such as *SpaGCN* utilize GCNs to integrate gene expression profiles, spatial coordinates, and histological imaging, thereby accurately identifying spatial domains and spatially variable genes [19]. Furthermore, advanced models like *GraphST* employ graph-based self-supervised contrastive learning to fully exploit spatial neighborhoods. These approaches significantly outperform traditional non-spatial clustering methods in deciphering tissue architecture and performing cell-type deconvolution [24].

### 2.3. Attention Mechanisms and Foundation Models

A significant paradigm shift in contemporary bioinformatics is the adaptation of attention mechanisms and Transformer architectures, originally pioneered in natural language processing, to transcriptomic analysis. Attention mechanisms are uniquely suited for modeling long-range dependencies, enabling networks to dynamically weigh the importance of specific genes and capture complex regulatory interactions across the entire transcriptome, irrespective of their linear genomic positions.

This architectural evolution has catalyzed the development of “foundation models” in single-cell biology. Through self-supervised pre-training on diverse datasets comprising tens of millions of cells, these models learn highly generalizable representations of cellular states and underlying Gene Regulatory Networks. A prominent example is Geneformer, a context-aware Transformer model that successfully facilitates zero-shot and few-shot predictions in network biology and disease target discovery [25]. Similarly, scGPT represents a substantial advancement toward a universal foundation model for single-cell multi-omics, demonstrating state-of-the-art performance in cell-type annotation, genetic perturbation prediction, and batch integration via generative pre-training [26].

### 2.4. Classical Machine Learning and Its Modern Adaptation

While deep learning architectures now dominate complex representation learning, classical machine learning algorithms, such as Random Forests (RFs), Support Vector Machines (SVMs), and k-Nearest Neighbors (KNNs), remain vital, albeit repositioned, components of the pipeline [5].

Rather than directly processing raw, high-dimensional sequencing counts, these traditional algorithms are now predominantly deployed in downstream stages following deep learning-based dimensionality reduction. For instance, KNN graphs are routinely constructed from dense latent representations to serve as the topological foundation for Louvain or Leiden clustering algorithms within standard toolkits like Seurat, ultimately defining robust cell subpopulations [27]. Furthermore, supervised ensemble models are frequently applied to these refined feature sets for targeted downstream tasks, including marker gene selection, clinical trajectory forecasting, and disease severity classification. By operating on lower-dimensional, denoised representations, classical ML algorithms continue to deliver the high interpretability, statistical rigor, and computational efficiency required for clinical and biological validation [16].

## 3. Application of Machine Learning in Transcriptomics Studies

The transition from classical machine learning to advanced deep learning in transcriptomics is not merely a shift in computational preference but a necessary response to the growing complexity, sparsity, and dimensionality of single-cell and spatial data [28]. Methodologically, this evolution follows a clear trajectory: early classical methods (such as PCA and simple clustering) provided initial dimensionality reduction; probabilistic generative models (like VAEs) emerged to rigorously address batch effects and dropout noise; subsequently, Graph Neural Networks (GNNs) were adopted to capture complex cell-cell and spatial topologies; and, most recently, Transformer-based foundation models have been introduced to leverage large-scale, self-supervised representation learning [4]. Understanding these methodological shifts is crucial for selecting the appropriate tool for specific biological inquiries.

**Figure 2:**
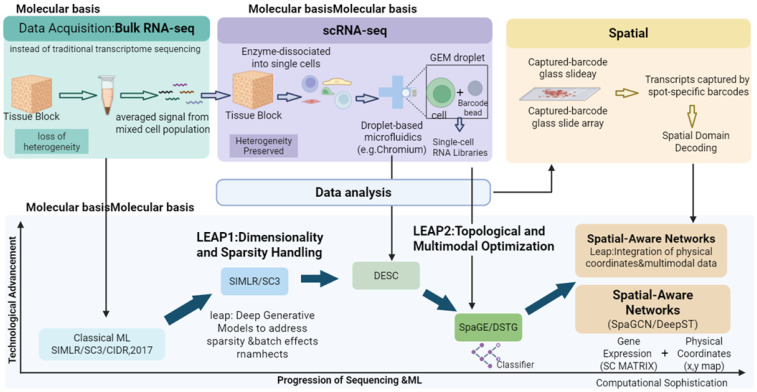
The methodological evolution and molecular basis of machine learning in transcriptomics. The diagram illustrates the biological progression of sequencing technologies alongside the corresponding computational leaps. Molecular Basics: The field evolved from Bulk RNA-sequencing (requiring tissue homogenization) to single-cell RNA-seq (requiring single-cell dissociation and droplet microfluidics), and finally to spatial transcriptomics (utilizing tissue sections on barcoded arrays to preserve physical morphology). Algorithmic Leaps: Corresponding to these technological shifts, computational methods have undergone significant leaps. Early single-cell analysis relied on classical statistical clustering (e.g., SC3, CIDR). As data sparsity grew, a methodological leap occurred toward deep generative architectures (e.g., DESC, gimVI) for robust representation learning. The emergence of spatial transcriptomics necessitated another critical leap: algorithms (e.g., SpaGE, DSTG) evolved to jointly optimize gene expression embeddings alongside physical tissue topologies. Legend: Green solid/dashed boxes denote bulk RNA-seq milestones and methods; purple boxes represent single-cell RNA-seq algorithms; and yellow/orange boxes indicate spatial transcriptomics applications. Dashed boxes denote the formal proposition milestones of the respective technologies.

Presently, the field of transcriptomics is broadly categorized into bulk RNA-sequencing, single-cell RNA-sequencing (scRNA-seq), and spatial/spatiotemporal transcriptomics. Figure 2 illustrates the parallel biological development of these sequencing technologies alongside their corresponding machine learning milestones. While high-throughput bulk RNA-seq became widely established around 2008–2009 [29], driving initial improvements in quantification efficiency, the advent of scRNA-seq in 2009 fundamentally shifted the analytical paradigm toward single-cell resolution. Subsequently, the formal proposition of spatial transcriptomics in 2016 introduced critical physical coordinates, enabling researchers to decode spatiotemporal gene expression dynamics within intact tissue architectures.

### 3.1. Machine Learning for Transcriptome Sequencing

While initial RNA-sequencing (RNA-seq) protocols emerged around 2008, their revolutionary impact on transcriptomics was comprehensively established by 2009 [29]. This technology relies on high-throughput sequencing to quantify RNA sequences and expression levels within specific cells, tissues, organs, or physiological states [30]. As the earliest and most widely adopted omics technology, bulk RNA-seq is essential for elucidating biological phenomena and the transcriptional regulatory mechanisms underlying complex phenotypes. However, the exponential growth of high-throughput sequencing data poses significant analytical challenges [31]. In this context, machine learning (ML) has emerged as a powerful analytical tool, injecting new vitality into bioinformatics. ML is now applied across both upstream and downstream stages of RNA-seq analysis to process high-dimensional datasets, automatically extract robust features, alleviate computational burdens, and enhance analytical automation.

From a methodological perspective, it is critical to highlight which specific ML models are designed for bulk data. Because bulk RNA-seq averages signals across millions of cells, the resulting data matrices are typically dense, continuous, and free from the extreme sparsity characteristic of single-cell data. Consequently, classical supervised ML algorithms, such as Support Vector Machines (SVMs), Random Forests (RFs), and gradient boosting (e.g., XGBoost), are specifically well-suited for bulk transcriptomics. These decision tree and margin-based methods excel at handling the “small sample, high feature” (p >> n) datasets typical of clinical cohorts, providing highly interpretable biomarker selection and disease classification. In contrast, applying massive deep neural networks directly to bulk expression data often leads to severe overfitting due to insufficient independent sample sizes.

**Figure 3:**
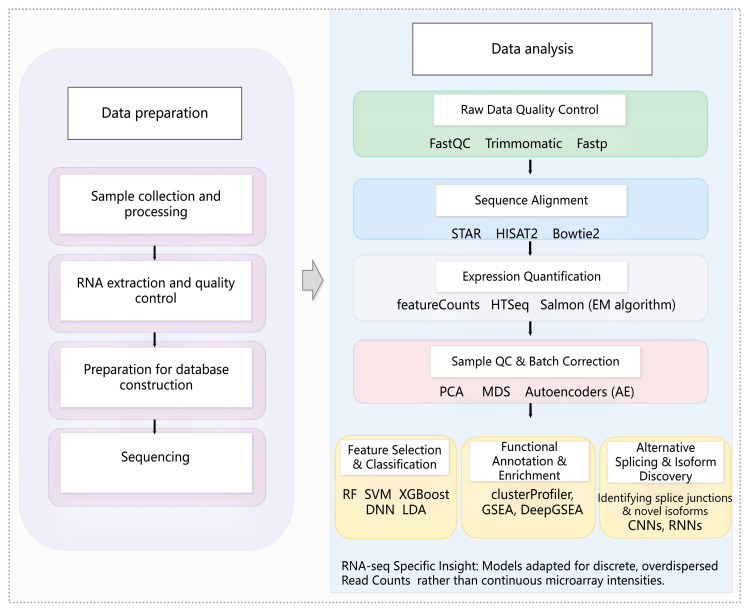
Flow of the transcriptome sequencing process. The left shows the transcriptome data extraction process and the transcriptome data analysis process on the right. Machine learning is mainly used in the analysis of transcriptome data.

A standard RNA-seq pipeline encompasses both experimental and computational phases, with the latter broadly divided into raw data quality control (QC), reference genome alignment, expression quantification, differential expression analysis, and functional annotation [9] (Figure 3). Traditionally, the read alignment step—matching short sequences to a known reference genome—does not directly utilize ML. Instead, it relies on specialized heuristic tools, such as Bowtie, STAR, HISAT2, and BWA. However, ML algorithms are predominantly applied in the subsequent preprocessing and downstream stages. During QC, algorithms like principal component analysis (PCA) and Autoencoders (AEs) can automatically identify outliers and technical noise, thereby ensuring data integrity for subsequent analyses. Furthermore, traditional normalization metrics (e.g., RPKM, FPKM, and TPM) adjust for sequencing depth but can introduce systematic biases. Because classical statistical methods often assume specific data distributions (e.g., normal distributions) that empirical transcriptomic data may violate, ML frameworks offer a robust alternative. They adapt to complex, non-linear data structures without strict parametric assumptions, ultimately reducing human bias and yielding more accurate gene expression estimates.

In downstream applications, while PCA [11] is routinely utilized for exploratory dimensionality reduction and variance visualization, supervised algorithms like linear discriminant analysis (LDA) and RF [10] are deployed for differential expression analysis. These methods efficiently select highly discriminative genes from massive candidate pools, providing crucial insights into biological processes and disease mechanisms. Finally, to annotate the biological functions of these transcripts, supervised ML algorithms like SVM [14,32] can accurately predict and classify genes with unknown functions based on established functional networks, significantly improving overall annotation efficiency.

Beyond enhancing sequence alignment and quantification, machine learning algorithms excel at processing transcriptomic profiles to predict clinical outcomes and screen for key driver genes [33]. Crucially, this classification capability enables ML frameworks to navigate qualitative or categorical decisions that cannot be represented by continuous variables alone. Such applications typically involve discrete biological classifications, including assigning cellular identities (e.g., T-cell versus B-cell), diagnosing qualitative disease states (e.g., healthy versus tumor), or predicting binary clinical responses (e.g., responder versus non-responder to targeted therapies). By classifying sample subtypes and predicting disease progression, these tools significantly advance the development of precision medicine and personalized therapy.

The application of supervised ML to disease classification has a rich history. For instance, as early as 2007, supervised models were applied to legacy microarray data to identify gene expression patterns distinguishing Parkinson’s disease (PD) patients from healthy controls [34]. More recently, ML algorithms such as k-Nearest Neighbors (KNNs) [35] and SVM [36] have been deployed on modern transcriptomic datasets to discover robust PD biomarkers. Furthermore, the SVM-core NetWAS algorithm [37] leverages transcriptomic functional networks to enhance the interpretation of genome-wide association study (GWAS) results, identifying phenotype-associated genes more effectively. In the realm of oncology, comprehensive evaluations of ML methodologies applied to next-generation sequencing (NGS) data have demonstrated exceptional efficacy in predicting cancer tissue of origin (TOO) [38,39]. These NGS-based predictive models achieved high prediction accuracies (73–99%), substantially outperforming traditional microarray-based techniques (54–100% [40]) and demonstrating immense potential for diagnosing carcinomas of unknown primary origin.

To mitigate the inherent transcriptomic challenges of data overfitting and extreme sensitivity to biological noise, rigorous algorithmic benchmarking is often essential in biomarker discovery [41]. For example, in autoimmune research, researchers utilized PCA and t-SNE for dimensionality reduction, followed by SVM and Random Forest (RF) models, to predict rheumatoid arthritis (RA) activity and treatment response. These models successfully identified highly predictive genes, such as *MGAT5*, *TNFSF10*, *ACAT1*, *CEBPB*, and *MMEL1*, whose expression levels strongly correlate with RA pathophysiology. Similarly, a comprehensive caret-based ML framework evaluated 13 distinct algorithms (including decision trees, Lasso/Elastic Net, GBM, and NNET) to discover radiation biomarkers in mouse renal tissues [42]. Following extensive optimization, the KNN algorithm emerged as the superior model, effectively classifying absorbed radiation doses and identifying seven novel biomarker candidates (e.g., *Brf2*, *Ccng1*), thereby circumventing the high computational overhead of previous genetic algorithm hybrid methods [43]. In infectious disease research, an evaluation of logistic regression, Naive Bayes, SVM, and XGBoost models for predicting COVID-19 severity determined XGBoost to be the most accurate [44]. Crucially, by integrating SHapley Additive exPlanations (SHAP), researchers could biologically interpret the model’s decision-making process, identifying key genes (e.g., *COX14*, *LAMB2*, *DOLK*) and clinical features (e.g., absolute neutrophil count, viremia categories) as severity drivers. These interpretable ML outputs provide clinicians with highly actionable insights into early disease trajectories. Detailed benchmarking parameters and validation metrics for these studies are summarized in Table 1.

### 3.2. Machine Learning for Single-Cell Transcriptome Sequencing

Before delving into specialized deep learning architectures, it is essential to establish the standard single-cell RNA-seq (scRNA-seq) analytical workflow. Currently, routine scRNA-seq analysis is predominantly driven by two comprehensive, industry-standard frameworks: Seurat [27] in the R (4.6.0) ecosystem and Scanpy [45] in Python (3.14). A conventional pipeline typically progresses through six highly standardized steps: (1) quality control (QC) to filter out dead cells and multiplets; (2) normalization and feature selection to identify highly variable genes; (3) linear dimensionality reduction (typically PCA), followed by non-linear visualization (e.g., UMAP or t-SNE); (4) data integration and batch correction to remove technical confounding factors; (5) graph-based clustering (e.g., Louvain or Leiden algorithms); and, finally, (6) cell-type annotation using established marker genes.

Unlike bulk data, single-cell transcriptomics introduces severe mathematical challenges: extreme sparsity, “dropout” events (zero-inflation), and complex, non-linear developmental manifolds. Classical linear tools and decision trees are inherently insufficient to handle this degree of biological and technical noise. Consequently, deep generative models (such as VAEs) and Transformer-based architectures have been specifically adapted for single-cell data. These neural network-based methods are uniquely capable of learning robust, low-dimensional latent spaces that inherently impute missing dropout values, seamlessly correct massive batch effects, and enable zero-shot cell-type annotations across diverse tissues, establishing them as the indispensable core of modern scRNA-seq analysis.

Understanding this baseline pipeline is critical for clarifying precisely where advanced machine learning models intersect with standard workflows. For instance, classical statistical methods like SC3 and CIDR aim to optimize the clustering and dimensionality reduction steps. In contrast, generative models like scVI and DESC primarily replace standard batch correction and integration protocols with robust probabilistic or deep embedded frameworks. Meanwhile, modern Transformer-based foundation models (such as scBERT or CelltypeGPT) are largely designed to automate and enhance the final cell-type annotation step via zero-shot or supervised learning. By conceptualizing these advanced architectures as modular upgrades to standard Seurat or Scanpy pipelines, researchers can design more robust, task-specific analytical strategies.

As the fundamental units of life, organisms comprise a diverse array of cells that undergo developmental differentiation to form complex tissues and organs with distinct lineages and specialized functions. Understanding biological systems at single-cell resolution is essential for elucidating the altered molecular mechanisms underlying various diseases [46]. While early methods for single-cell molecular analysis emerged in the late 20th century, the advent of true single-cell RNA-sequencing (scRNA-seq) in 2009 marked a revolutionary milestone [47]. Driven by continuous improvements in experimental protocols and high-throughput sequencing platforms, the scale of scRNA-seq experiments has grown exponentially—from profiling a single cell in 2009 to exceeding one million cells per study by 2017 [47]. Today, the acquisition of scRNA-seq data is ubiquitously employed across diverse fields of biology and precision medicine [38,48,49].

Conventional bulk RNA-sequencing (bulk RNA-seq) typically profiles entire organs or tissue homogenates, yielding an averaged snapshot of global gene expression. This macroscopic approach inherently masks the profound spatial and temporal transcriptomic heterogeneity that exists among distinct cell subpopulations [50]. The emergence of scRNA-seq effectively resolves this limitation by distinguishing individual cell types and capturing independent, cell-specific expression profiles. As scRNA-seq technologies have rapidly matured, the primary research bottleneck has shifted from experimental data acquisition to the computational analysis of massive datasets. A standard scRNA-seq analytical pipeline typically encompasses raw data dimensionality reduction, denoising [51], and subsequent classification tasks—most notably, graph-based clustering [52] and cell-type annotation.

Cluster analysis is particularly pivotal, as it reveals cellular heterogeneity and developmental diversity by delineating specific cell types and states. However, compared to bulk RNA-seq, scRNA-seq datasets present unique and severe mathematical challenges. A defining obstacle is extreme data sparsity, characterized by the prevalent “dropout” phenomenon (zero-inflation), where expressed transcripts fail to be detected due to minute starting RNA quantities [53,54]. Furthermore, technical artifacts inevitably introduce batch effects when integrating datasets generated across different protocols, platforms, or biological replicates [55]. Compounding these issues is a high degree of biological and technical noise, including stochastic transcriptional bursting, mRNA degradation, and inadequate sequencing depth [56]. Because many features (genes) in scRNA-seq matrices are zero or near-zero, this pervasive noise obscures subtle transcriptomic differences between closely related cell states. Finally, the inherently high-dimensional nature of these datasets [57] exponentially increases the computational complexity of analysis, underscoring the absolute necessity for advanced, noise-tolerant machine learning algorithms.

To robustly identify cell types across novel samples, highly accurate computational methods are essential. In recent years, numerous machine learning classification algorithms specifically tailored for single-cell data have been proposed [58,59,60,61,62,63,64,65], significantly advancing data processing capabilities. Both supervised algorithms (such as SVM and logistic regression) and unsupervised approaches (like K-means clustering) are routinely deployed for cell-type identification. For instance, CIDR [66] tackles data sparsity through dropout-aware implicit imputation to adjust cell–cell dissimilarity matrices, which are subsequently processed using principal coordinate analysis (PCoA)—a classical multidimensional scaling technique—followed by hierarchical clustering, rather than relying on standard PCA. SC3 [67] integrates multiple distance metrics (Euclidean, Pearson, and Spearman) and transformations (PCA and graph Laplacian), executes parallel K-means clustering across multiple dimensions ($d$), and performs hierarchical clustering on the resulting consensus matrix to effectively decode cellular heterogeneity. SIMLR [68] employs multiple kernel learning (MKL) to capture complex intercellular similarities based on gene expression profiles. Although these methods have substantially improved single-cell clustering efficiency, they often struggle to provide optimal solutions when processing highly sparse scRNA-seq matrices. Furthermore, many of these classical algorithms incur prohibitive computational and storage overhead, limiting their scalability to modern, million-cell atlases.

To overcome these limitations, the integration of deep learning with scRNA-seq analysis has emerged as a transformative frontier in the life sciences. Numerous innovative deep neural network architectures have been developed, providing powerful new paradigms for single-cell data representation. For example, DESC [69] is built upon a stacked Autoencoder that utilizes mean squared error (MSE) reconstruction loss paired with Kullback–Leibler (KL) divergence-based self-training within a deep embedded clustering (DEC) framework. Notably, it iteratively reduces batch effects implicitly without requiring prior batch labels or explicit zero-inflated negative binomial (ZINB) likelihood assumptions. Similarly, scBERT [70] strictly employs a Performer-based architecture leveraging FAVOR+ linear attention to efficiently process long gene sequences (over 16,000 genes). It bins gene expression values, embeds gene identities via gene2vec, and utilizes masked expression value prediction during its pre-training phase. Other models focus on specific clustering optimization: ADCluster [71] simultaneously performs anomaly detection and cluster analysis to isolate outliers while grouping normal cells, whereas scCAEs [72] utilizes a convolutional Autoencoder paired with a soft K-means deep embedding layer to identify latent cellular subpopulations. Furthermore, scDCCA [73] integrates denoising Autoencoders (DAEs) with contrastive learning modules to extract robust biological features.

Despite these advancements, many foundational deep learning algorithms do not explicitly model gene–gene regulatory mechanisms or intrinsic cellular topologies. Addressing this, the *DeepGRNCS* [74] framework utilizes deep neural networks to predict target gene expression based solely on the expression of transcription factors (TFs), bypassing the need for prior topological information in Gene Regulatory Network (GRN) inference. When benchmarked against existing bulk-based (e.g., PIDC, GENIE3) and single-cell-specific (e.g., NETI2, JEGN) GRN inference tools using simulated datasets, *DeepGRNCS* demonstrated superior clustering and network prediction performance. Its subsequent application to real scRNA-seq tumor datasets effectively mapped complex intra-tumoral heterogeneity. Finally, leveraging the power of large language models (LLMs), the CelltypeGPT [75] software package functions as a GPT-4 API-based prompting wrapper. Accessible as an R package, it efficiently annotates cell types by taking lists of marker or differentially expressed genes as input, operating as an intelligent annotation conduit rather than a standalone pre-trained foundation model.

Driven by the success of self-supervised representation learning, the single-cell community has witnessed an explosion of foundational models pre-trained on tens of millions of cells. The defining advantage of these Transformer-based foundation models lies in their robust cross-dataset generalization. Beyond Geneformer and scGPT, recent architectures such as scFoundation [21], UCE (Universal Cell Embedding) [76], and CellPLM [77] have significantly advanced cross-tissue and cross-species zero-shot annotations by learning universal cellular representations, allowing them to overcome batch effects and accurately perform cell-type annotation across novel, unseen biological contexts. Furthermore, beyond standard clustering and annotation, this generative capacity has unlocked entirely new predictive frontiers, most notably in perturbation prediction. Early VAE-based models like scGen [78] and CPA [79] laid the groundwork, while more recent models like GEARS [80] integrate Graph Neural Networks and deep learning to accurately simulate the transcriptional outcomes of combinatorial genetic perturbations in silico. This significantly accelerates drug discovery and functional genomics without the immediate need for exhaustive laboratory screening.

It should be noted that most of the existing algorithms rely on gene expression information during characterization learning while not implementing sharing topological information between cells. In order to further capture the complex relationships between cells and their intrinsic properties, several algorithms based on Graph Neural Networks (GNNs) have been proposed. The GNN can reveal the connections between the target node and its surrounding nodes, thereby enhancing the representation of the node features. For example, scGAC [81] specifically relies on Graph Attention Networks (GATs) coupled with Network Enhancement for graph denoising and DEC-style self-training. scDFC [82] uses attribute information and structural information based on attention mechanisms to precisely construct intercellular maps to address complex biological situations. GNN can capture and represent complex higher-order structural relationships between cells, but existing GNN-based clustering methods easily map different classes of nodes to similar representations during encoding and cannot effectively distinguish different types of cell. The deep learning framework scZAG [83] combines APPNP-style personalized PageRank propagation with a ZINB Autoencoder, graph contrastive learning, and self-optimizing clustering to map intricate cellular relationships. The iterative propagation mechanism of APPNPGCN helps the scZAG model to better capture the complex relationships between cells. By comparing with other state-of-the-art scRNA-seq clustering methods in 10 real datasets, the results show that scZAG clustering performs better and can more effectively distinguish and isolate various cell populations. Another deep framework, DCRELM [84], is not a conventional single-layer classifier; rather, it is a hybrid deep model that integrates Extreme Learning Machine (ELM)-based random mapping, dual-view perturbation, Autoencoder fusion, and triplet self-supervised learning to stabilize feature representation for clustering. The learning machine performs highly by clustering comparison with the six latest single-cell clustering methods on 12 real scRNA-seq datasets.

While complex deep learning architectures have gained significant traction, a scientifically balanced perspective requires acknowledging that standard, highly optimized baseline methods remain the workhorses of practical single-cell analysis. For batch correction and data integration, classical frameworks often perform exceptionally well. Methods such as Harmony [85], Seurat v3/v4 (utilizing integration anchors and Weighted Nearest Neighbor (WNN) analysis), MNN/fastMNN (Mutual Nearest Neighbors) [86], LIGER (based on integrative non-negative matrix factorization) [87], and BBKNN are widely established standards. Similarly, for automated cell-type annotation, classical machine learning approaches frequently provide robust, lightweight, and highly interpretable alternatives to deep learning. Widely used standard tools, including SingleR [88], scmap, scPred, CellTypist, and Azimuth, leverage reference datasets and regularized classifiers to achieve highly accurate annotations, often serving as critical baselines against which newer foundation models must be rigorously benchmarked. Among deep generative models, the Variational Autoencoder (VAE) framework has become truly foundational for single-cell analysis. The introduction of scVI [17] established a robust generative probabilistic model that serves as the baseline for numerous modern pipelines, efficiently handling dropout, batch effects, and differential expression. The scvi-tools [22] ecosystem has since expanded into a comprehensive framework, providing highly specialized architectures: scANVI [89] leverages cell-type labels for semi-supervised annotation, totalVI [90] facilitates the integration of multimodal data (such as joint RNA and surface protein measurements), and scArches [23] utilizes transfer learning to map query datasets onto massive reference atlases without the computationally prohibitive need to retrain the entire model.

Further studies of individual cells will help in the personalized medicine field for a deeper understanding of the underlying processes in various developmental physiologies and disease systems. One study proposed DeepGSEA [91], which utilizes a prototype-based interpretable deep neural network combined with classification-based significance testing to capture complex gene set distributions, distinguishing it from traditional ranked GSEA. Compared with commonly used existing methods, DeepGSEA has a high ability to capture gene profile differences between phenotype groups. DeepIMAGER [92] converts gene-pair co-expression patterns into image-like histograms, subsequently applying Convolutional Neural Networks (CNNs) for supervised Gene Regulatory Network (GRN) inference, continuing the lineage of models like CNNC. Tested in six real scRNA-seq datasets, DeepIMAGER showed superior performance in comparison with 10 popular GRN inference tools. DeepIMAGER was applied to the scRNA-seq dataset of multiple myeloma and successfully detected a potential GRN of the TF of *RORC*, *MITF* and *FOXD2* in MM dendritic cells. Detailed test information is given in Table 2.

It is crucial to emphasize that advanced downstream analyses, such as Gene Regulatory Network (GRN) inference and cell–cell communication analysis, should not be simply equated with a single class of machine learning methods. Rather, they are complex, multi-step analytical frameworks that rely heavily on prior biological assumptions and discrete model choices. For instance, GRN inference typically requires preprocessing steps to handle dropout, followed by the integration of prior knowledge (such as transcription factor binding motifs) to constrain the search space. Within this framework, machine learning models (such as the DNNs in *DeepGRNCS* or the CNNs in DeepIMAGER) do not operate as monolithic solutions; instead, they contribute to specific components, such as performing non-linear regression for link prediction or estimating the statistical weights of regulatory edges. Similarly, cell–cell communication analysis is fundamentally anchored in curated ligand–receptor interaction databases. When advanced algorithms (like spatially aware networks) are applied to this domain, their specific role is usually to calculate interaction probabilities, infer spatial proximity constraints, and denoise the underlying expression data. By recognizing machine learning as a specialized computational engine within a broader biological systems framework, researchers can avoid the “black box” trap and make more informed, interpretable analytical choices.

In summary, selecting an analytical framework for single-cell transcriptomics requires carefully balancing computational cost with biological necessity. While Variational Autoencoders (VAEs) excel at probabilistic integration and batch correction, they often lack the explicit ability to model intricate spatial or communication networks. Conversely, Graph Neural Networks (GNNs) are highly specialized for topology and trajectory mapping but can be sensitive to graph construction parameters. Meanwhile, the recent surge of Transformer-based foundation models offers unprecedented capabilities for cross-tissue zero-shot annotation; however, they require massive computational resources and, as recent evaluations suggest [93,94], may not inherently outperform classical linear baselines in simpler, localized tasks. Therefore, future pipeline development must prioritize algorithmic efficiency and rigorous, task-specific benchmarking [95] rather than merely increasing model parameters.

### 3.3. Machine Learning for Spatial Transcriptome Sequencing

The evolution of transcriptomic technologies has progressed through three pivotal stages. Initially, bulk RNA-sequencing profiled massive admixtures of cells, capturing only average gene expression levels and fundamentally masking cell-specific transcriptional dynamics. The subsequent advent of single-cell transcriptomics extended this resolution to individual cells, enabling researchers to decode organ function and disease progression at unprecedented cellular and subcellular levels [96]. However, traditional scRNA-seq requires the dissociation of solid tissues into single-cell suspensions, inevitably destroying the native spatial coordinates of each cell [97]. Preserving this spatial context is imperative for deeply understanding tissue structure–function relationships, localized cell–cell interactions, and microenvironmental regulation. While early in situ hybridization methods (e.g., FISH) laid the pioneering groundwork for spatial mapping, the formal term “spatial transcriptomics” (ST) was coined in 2016 when Lundeberg and colleagues introduced the first high-throughput, array-based technique for capturing RNA in situ [18]. By integrating ST with conventional scRNA-seq and other multi-omic modalities, researchers can comprehensively map cellular heterogeneity back to precise histological coordinates, offering profound insights into disease pathogenesis and targeted therapeutic design. Recognizing its transformative capacity—driven by continuous improvements in cellular throughput, transcript capture efficiency, and spatial resolution—Nature Methods crowned spatially resolved transcriptomics as the “Method of the Year” in 2020 [98].

Methodologically, scRNA-seq and ST data are highly complementary. While ST inherits several technical limitations characteristic of scRNA-seq—most notably low capture efficiency and severe dropout rates—it introduces entirely new dimensions of complexity. Although many computational pipelines originally developed for scRNA-seq can theoretically be adapted for ST analysis [20], such direct translations must be approached with caution. ST transcript pools do not necessarily follow the same statistical distributions as scRNA-seq data, primarily because standard ST capture spots often contain mixtures of multiple cells rather than pure, isolated single cells. Consequently, standard single-cell analytical assumptions must be rigorously reassessed. Furthermore, the sheer volume and multidimensional nature of ST data present formidable computational bottlenecks, underscoring the necessity for advanced machine learning interventions. Detailed benchmarking of these ML applications is summarized in Table 3.

What fundamentally distinguishes spatial transcriptomics from both bulk and single-cell data is the introduction of a new mathematical dimension: physical (x,y) coordinates and tissue histology. Algorithms designed exclusively for scRNA-seq treat cells as isolated entities and cannot process physical topology. To bridge this gap, Graph Neural Networks (GNNs) and spatially aware Convolutional Neural Networks (CNNs) were specifically designed for spatial data. By treating individual capture spots as nodes and their physical or histological proximity as edges, these specialized architectures can jointly optimize gene expression embeddings alongside physical tissue morphology, enabling the precise decoding of spatial domains and cell–cell communication microenvironments.

To enhance data resolution and overcome the inherent sparsity of spatial transcriptomics (ST), computational imputation—driven by deep learning, dimensionality reduction, or other machine learning algorithms—is frequently employed to predict missing gene expression profiles by leveraging matched single-cell RNA-sequencing (scRNA-seq) references. For example, the VAE-based generative model gimVI [99] integrates ST data with unlabeled scRNA-seq matrices. By utilizing conditional distributions, it effectively accounts for platform-specific technical effects and seamlessly processes multi-batch datasets. Alternatively, SpaGE [100] employs principal component analysis (PCA) and singular-value decomposition (SVD) to project both modalities into a shared latent space, subsequently utilizing *k*-Nearest Neighbor (KNN) interpolation to impute unmeasured genes. SpaGE demonstrated superior predictive accuracy over contemporary baselines (Seurat, gimVI, and LIGER) across diverse regions of the mouse brain. Furthermore, it exhibits exceptional scalability, significantly reducing computational and memory overhead when applied to massive spatial cohorts, such as MERFISH datasets comprising over 60,000 spatial measurement units. Building upon this shared-space paradigm, the Autoencoder-based model stPlus [101] leverages weighted KNN clustering to forecast spatial gene expression. Empirical evaluations utilizing Spearman correlation coefficients indicated that stPlus consistently outperformed mainstream predictive methods. Another prominent deep learning framework, Tangram [102], aligns scRNA-seq or single-nucleus RNA-seq (snRNA-seq) profiles directly to spatial coordinates. By correlating these profiles with matched histological and anatomical imaging from the same sample, Tangram resolves transcriptome-wide spatial expression at true single-cell resolution, effectively overcoming the inherent throughput limitations of primary ST platforms.

Beyond gene imputation, machine learning is indispensable for spatial deconvolution—the computational estimation of discrete cell-type proportions within macroscopic ST capture spots. For instance, SPOTlight [103] integrates ST and scRNA-seq data via non-negative matrix factorization (NMF). Initialized with cell-type marker genes and non-negative least squares (NNLS), it deconvolves ST capture locations to map complex tissue architectures, successfully reconstructing the hierarchical layers of the mouse brain and detailing clinically relevant immune microenvironments in pancreatic adenocarcinoma. However, classical NMF approaches often fail to incorporate the intrinsic spatial topology of neighboring spots. To address this, DSTG (Deconvoluting Spatial Transcriptomics Data through Graph-based Artificial Intelligence) [104] deploys a semi-supervised graph convolutional network (GCN) that explicitly incorporates spatial neighborhood topologies alongside scRNA-seq references, significantly enhancing deconvolution accuracy in heterogeneous tissues. Alternatively, SpatialDWLS [105] utilizes dampened weighted least squares (DWLS) on enriched gene signatures to estimate local cell-type proportions, rigorously accounting for basal expression variances across different lineages. It has demonstrated exceptional accuracy and practicality across both simulated platforms and real Visium datasets. Similarly, RCTD (Robust Cell-Type Decomposition) [106] applies maximum likelihood estimation (MLE) to scRNA-seq profiles to resolve ST mixtures, introducing critical statistical corrections for cross-platform sequencing biases and enabling the precise mapping of subtle cellular subtypes.

Expanding beyond pure deconvolution, the multimodal deep learning framework DeepST [107] jointly embeds spatial coordinates, histological morphology, and gene expression to delineate distinct spatial domains. DeepST not only corrects massive batch effects but also generalizes across diverse ST platforms (e.g., MERFISH, Slide-seq, Stereo-seq). Notably, when applied to breast cancer ST datasets, it identified highly heterogeneous intra-tumoral regions previously undetected by conventional transcriptomic analyses. Most recently, the integration of Transformer-based architectures with spatial awareness has catalyzed the emergence of spatially resolved foundation models. For example, Nicheformer leverages spatially contextualized, self-supervised learning to construct comprehensive representations of tissue microenvironments, thereby unlocking unprecedented nuances in cell–cell communication modeling.

### 3.4. Trajectory Inference and Cellular Dynamics

A critical pillar of single-cell analysis, alongside clustering and integration, is the modeling of cellular dynamics, state transitions, and differentiation trajectories. Traditional trajectory inference methods construct pseudotime orderings to map developmental lineages; foundational tools in this space include the Monocle suite (up to Monocle 3) and PAGA (Partition-based Graph Abstraction), which efficiently map complex, disconnected topologies within massive datasets.

The conceptualization of RNA velocity [109] revolutionized this domain by utilizing the ratio of spliced to unspliced transcripts to predict the future transcriptional states of individual cells. This was rapidly advanced by machine learning frameworks such as scVelo, which introduced a likelihood-based dynamical model to resolve transient cellular states and complex kinetics. Building upon this foundation, sophisticated tools like CellRank and its iteration CellRank 2 [110] combine RNA velocity with robust graph-based machine learning (e.g., Markov chains) to uncover probabilistic cell fate decisions. These algorithms effectively map complex multi-lineage trajectories, seamlessly identifying initial, terminal, and intermediate cellular states.

### 3.5. Machine Learning for Spatiotemporal Transcriptomics

While single-cell and spatial transcriptomics have fundamentally resolved cellular heterogeneity and spatial organization, respectively, early iterations of these technologies lacked a temporal dimension. Spatiotemporal transcriptomics has rapidly emerged as a transformative technology that concurrently quantifies gene expression, precisely maps cellular spatial coordinates, and tracks these variables across discrete developmental or disease time points. By sequencing consecutive tissue sections across distinct temporal stages at single-cell resolution, researchers can dynamically trace the spatial trajectories of specific cell lineages. This multidimensional approach facilitates the discovery of rare cell types and elucidates the regulatory mechanisms governing cell differentiation and organogenesis [111]. Highlighting its immense research potential, spatiotemporal omics was prominently featured as a transformative technology by *Nature* in 2022 [112], coinciding with the establishment of the international Spatio-Temporal Omics Consortium (STOC) in Shenzhen, China.

As a high-throughput methodology integrating space and time, spatiotemporal transcriptomics is being extensively applied across diverse biological kingdoms. In animal models, it is pivotal for tracking tumor microenvironment evolution, mapping nervous system development, and characterizing immune responses. In botany, its applications span plant organ development, morphological evolution, and stress resistance. Because plant cells possess unique, rigid structures, such as cell walls, vacuoles, and chloroplasts, standard cell segmentation and clustering algorithms initially struggled to perform accurately. Recently, however, ML algorithms utilizing specialized convolutional architectures and deep watershed models are being explicitly adapted to recognize plant-specific morphological boundaries, substantially expanding spatiotemporal applications in plant biology. Similarly, while applications in microbiology remain nascent, deep learning networks are increasingly deployed to model host–pathogen spatiotemporal interactions. For instance, spatially aware graph algorithms have demonstrated strong potential in tracking infectious diseases, enabling the decoding of virus-specific host responses and the precise spatial resolution of microenvironmental damage caused by SARS-CoV-2 [113,114].

**Figure 4:**
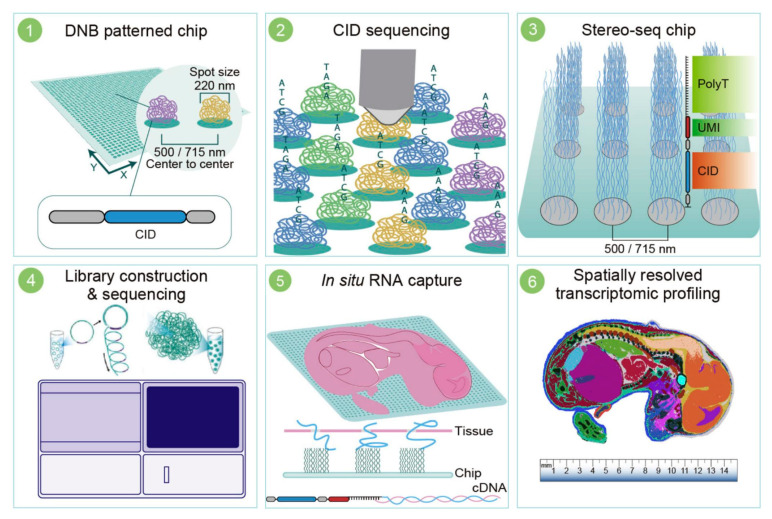
Stereo-seq pipeline (Adapted from Chen et al. [115] under the terms of the Creative Commons CC-BY license.) Step 1, design of the DNB mode chip. DNB libraries containing random barcodes were designed and their templates were deposited on the chip. The chips were sequenced in situ using primers to determine the spatial coordinates (CID) of each DNB. Step 2, in situ sequencing to determine the spatial coordinates of uniquely barcoded oligonucleotides. Step 3, preparation of capture probes by ligating the UMI-polyT containing oligonucleotides to each spot. Step 4, in situ RNA capture from tissue. Step 5, Libraries were constructed and sequenced. The amplified cDNA was used to construct the sequencing libraries and was sequenced together with the CID. Step 6, Data analysis of the sequencing data was performed using the computational analysis tool.

A monumental milestone in spatiotemporal omics was the 2021 introduction of Stereo-seq, developed by BGI-Research (Shenzhen, China) in conjunction with international collaborators. Stereo-seq is a pioneering spatial transcriptomic technology that achieves unprecedented subcellular resolution (500 nm) coupled with a centimeter-scale panoramic field of view (up to 13 cm × 13 cm). By integrating DNA nanoball (DNB) patterned arrays with in situ RNA capture technologies, Stereo-seq decodes the spatiotemporal dynamics of gene expression across massive tissue areas without sacrificing high throughput. This technology has demonstrated exceptionally broad applicability, having been successfully utilized to map the adult mouse brain and track sagittal sections of mouse embryos across various developmental stages, thereby validating its adaptability for profound biological discovery. A detailed workflow of the Stereo-seq methodology is illustrated in Figure 4 [115].

To alleviate the analytical bottlenecks associated with the highly complex, multidimensional spatial data generated by Stereo-seq, BGI-Research developed the Spatial Analysis Workflow (SAW). This comprehensive software suite integrates multiple machine learning algorithms to streamline data processing and assist researchers in deciphering intricate biological structures. The SAW pipeline encompasses a complete suite of standard procedures, including raw data preprocessing, quality assessment, batch effect correction, cell clustering, cell-type identification, and spatial network analysis. Notably, during the preprocessing phase, the STCellbin [108] module deploys the deep learning-based universal segmentation algorithm, Cellpose 2.0, to achieve highly accurate, morphology-aware cell segmentation.

Ultimately, the defining methodological distinction between single-cell and spatial transcriptomics analysis lies in the incorporation of physical coordinates. While traditional single-cell machine learning focuses exclusively on transcriptional similarity, spatial algorithms, particularly those leveraging spatially aware GNNs and spatial Transformers, must jointly optimize gene expression embeddings alongside physical tissue topology. A critical synthesis of these methodologies reveals a clear consensus: integrating multimodal inputs [4] through hybrid neural architectures represents the most robust computational strategy for uncovering genuine tissue microenvironments [4,116].

### 3.6. Machine Learning for Multimodal Data Integration

Modern transcriptomics increasingly relies on multimodal data to capture comprehensive cellular states. Machine learning is uniquely positioned to bridge scRNA-seq with epigenomic (e.g., scATAC-seq), proteomic (e.g., CITE-seq), and histological imaging modalities. Early probabilistic frameworks, such as totalVI [90], established the foundation by enabling the joint modeling of RNA and surface proteins. More recently, deep learning has driven sophisticated cross-modality translations. For instance, the BABEL algorithm utilizes a deep neural network (DNN) to integrate chromatin profiles with transcriptomes, accurately predicting single-cell gene expression directly from chromatin accessibility (scATAC-seq) data [117]. Similarly, Hist2ST [118] is a specialized deep learning model capable of generating spatial transcriptomic maps directly from standard histological images. Furthermore, ConGI [119] employs CNN- and MLP-based architectures via contrastive learning to seamlessly integrate histopathological imaging with spatial transcriptomics. This approach effectively decodes spatial domains by learning common cross-modal embeddings while rigorously isolating modality-specific noise.

## 4. Existing Challenges and Future Prospects

Machine learning (ML) techniques have been applied to transcriptomic research for nearly two decades, fundamentally focusing on extracting biological insights from RNA sequences, epigenetic modification sites, and gene expression profiles. The massive scale and inherent complexity of transcriptome sequencing data pose significant challenges for traditional analytical methods, particularly regarding high-throughput processing, pattern recognition, and multi-omic integration. However, these bottlenecks can be navigated much more efficiently and robustly utilizing ML technologies. While ML has not entirely replaced classical statistical frameworks, the exponential increase in ML-focused transcriptomic publications underscores its profound and expanding potential. Indeed, ML has revolutionized transcriptomic data analysis, not only by enhancing computational efficiency and analytical depth but also by providing unprecedented perspectives for biological discovery and clinical application. ML offers several distinct advantages: it automates the processing of massive datasets, significantly reduces the need for manual intervention, and seamlessly integrates principles from biology, statistics, and computer science. By autonomously learning data distributions, extracting latent features, and uncovering hidden biological relationships, ML fundamentally improves both processing efficiency and diagnostic accuracy. Furthermore, ML algorithms excel at integrating transcriptomic data across disparate platforms and experimental conditions, effectively mitigating batch effects to yield highly comprehensive interpretations. Particularly with the rapid evolution of deep learning (DL), a massive influx of novel neural network architectures has been successfully adapted for transcriptomics.

Despite these remarkable advancements, the application of ML to transcriptomic data exposes several critical challenges, primarily stemming from the extreme diversity and biological complexity of the data. First, the high-dimensional nature of sequencing data frequently predisposes complex ML models to severe overfitting. Second, extreme class imbalances (e.g., highly variable sample sizes across different disease states or rare cell types) often bias models toward majority classes, complicating the training of stable algorithms on small or rare cohorts. Third, the profound spatial heterogeneity and dynamic temporal shifts inherent to cellular states require models capable of mapping 4D spatiotemporal trajectories—a formidable algorithmic hurdle. Furthermore, state-of-the-art ML models often require massive computational resources. Deep neural networks containing millions of parameters inherently function as opaque “black boxes,” making their biological decision-making processes difficult to understand or validate. To overcome this limitation, the integration of Explainable AI (XAI) techniques, such as SHAP (SHapley Additive exPlanations), LIME (Local Interpretable Model-agnostic Explanations), and GNNExplainer, is becoming essential to demystify these complex architectures and provide transparent, biologically meaningful feature attributions. Finally, the steep interdisciplinary learning curve poses a practical barrier; biologists and clinicians without extensive computational backgrounds may struggle to deploy these models effectively, hindering their ultimate translation into routine clinical practice. Consequently, future algorithmic development must prioritize high computational efficiency, rigorous interpretability, and low resource consumption.

Despite the rapid proliferation and substantial parameter scale of single-cell foundation models, their genuine utility in specific downstream tasks remains a subject of critical ongoing debate. Recent rigorous evaluations have suggested that in many zero-shot settings and specific batch-integration scenarios, these resource-intensive foundation models do not consistently outperform classical, simple statistical baselines. For example, critical analyses by Kedzierska et al. (2025) [120] demonstrated that basic logistic regression or non-deep-learning Nearest Neighbor approaches often match or even exceed the performance of pre-trained Transformers on out-of-the-box cell-type annotation and embedding tasks. These findings highlight a significant current challenge: while representation learning offers vast potential, there is an urgent need to establish standardized, rigorous benchmarking frameworks to ensure that the deployment of complex machine learning models translates to tangible biological utility rather than mere computational overhead.

## 5. Conclusions

Machine learning (ML) has demonstrated immense potential and catalyzed significant advancements within the field of transcriptomics. It consistently outperforms traditional analytical frameworks by excelling in high-throughput data processing, robust latent feature extraction, noise tolerance, and cross-dataset generalization. Nonetheless, formidable challenges persist. The extreme dimensionality of transcriptomic profiles, pervasive technical batch effects, the inherent “black-box” opacity of complex neural networks, and the substantial computational overhead required for model training continue to limit broader deployment. Addressing these bottlenecks will necessitate the rigorous refinement of current algorithms, prioritizing computational efficiency and robust interpretability. As spatial and single-cell sequencing technologies continue to evolve and data acquisition costs plummet, the full transformative capacity of ML in transcriptomics will be realized. Ultimately, these intelligent analytical pipelines will drive profound breakthroughs across precision medicine, agricultural genetics, fundamental biological sciences, and environmental protection.

## Figures and Tables

**Table 1:** Application of partial machine learning in transcriptome sequencing.

Assignment	Model Name	Machine Learning Algorithm	Validation (Methods, Data Set, Results)	Published Time	Reference
To evaluate the prediction accuracy of the samples in the training set	Not reported	KNN	The qPCR validation of 13 risk markers in two independent test sets showed that PD patients could be accurately identified based on these 13 markers, with a sensitivity of 90% and a specificity of 94%.	2012	[35]
Classifier trained to distinguish between PD patients and healthy controls	Not reported	SVM	On the independent test set, the model distinguished well between Parkinson’s disease patients and healthy controls (AUC of 0.74).	2017	[36]
Reordering of genes based on the connectivity patterns of tissue-specific functional networks	NetWAS	SVM	Application to hypertension GWAS data revealed that NetWAS could more effectively identify genes associated with hypertension, and that the genes identified by NetWAS were significantly enriched with functional annotations and drug targets related to hypertension.	2015	[37]
Identified genes that to distinguish different dose groups and tissue types as radiation biomarker candidates	caret-KNN	KNN	The KNN model achieved high accuracy (0.86) and K values (0.49,0.49) in both the training set samples and the test set, and high R^2^ values (0.97,0.99) in dose prediction.	2023	[42]
Predict the COVID-19 severity	Not reported	XGBoost	The RNA-seq data, clinical characteristics, and comorbidity information of 299 hospitalized COVID-19 patients were analyzed and compared. Compared with LR, Naive Bayes, and SVM, the XGBoost model performed the best in predicting COVID-19 severity with 95% accuracy and an AUC of 0.99.	2024	[44]

**Table 2:** Application of partial machine learning in the single-cell transcriptome.

Assignment	Model Name	Machine Learning Algorithm	Validation (Methods, Data Set, Results)	Published Time	Reference
Tackles sparsity through dropout-aware implicit imputation to adjust cell–cell dissimilarity for subsequent dimensionality reduction and clustering.	CIDR	PCoA (Principal Coordinate Analysis), Hierarchical clustering	Using simulation data and biological data, and compared with the standard PCA, t-SNE, ZIFA and RaceID algorithms, CIDR achieved good clustering results and ran time much faster than the other algorithms.	2017	[66]
Integrates multiple metrics and transformations to generate a robust consensus matrix for downstream hierarchical clustering.	SC3	Multiple distance metrics, PCA/graph Laplacian, Parallel k-means, Hierarchical consensus clustering	Using six published datasets compared with five commonly used scRNA-seq data clustering methods including tSNE + kmeans, pcaReduce, SNN-Cliq, SINCERA and SEURAT, SC3 had higher ARI values on all datasets and SC3 was able to identify rare cell types containing 1% or 10% cells.	2017	[67]
Learning the similarity measures between cells	SIMLR	Multi-kernel Learning, Rank Constraint, Graph Diffusion, K-means, t-SNE	Tested in human PBMC, mouse cortex and hippocampal datasets, compared with PCA + K-means, t-SNE + K-means and single-cell data analysis tool Seurat, showed higher ARI values and completed analysis of datasets containing hundreds of thousands of samples within minutes.	2017	[68]
Avoids explicit ZINB assumptions to iteratively reduce batch effects implicitly without requiring prior batch labels.	DESC	Stacked Autoencoder (MSE loss), KL-divergence self-training (DEC framework)	In the five datasets, compared with methods such as CCA, MNN, Seurat 3.0, scVI, BERMUDA, and scanorama, the ARI values of DESC were above 0.9, exceeding the remaining methods. And it can still maintain a high ARI when dealing with complex batch effects and large-scale datasets.	2019	[69]
Employs a Performer architecture to efficiently handle long gene sequences with masked expression-value prediction pretraining.	scBERT	Performer-based architecture (FAVOR+ linear attention), gene2vec	The scBERT was evaluated on six datasets, including cell type annotation, across batches, robustness on organ dataset, ability to find new cells, performance on large-scale and hyperparameter sensitivity on six datasets. In the cross-organ dataset, scBERT performance was comparable to the remaining methods, and in five additional ways, scBERT accuracy and macro F1 scores outperformed the other algorithms.	2022	[70]
Iterative optimization, merging microclusters with similar structure, yields high-quality clustering results and effective low-dimensional features	ADCluster	Deep Embedded Clustering	Testing with 11 real and 5 simulated scRNA-seq datasets showed that ADClust has superior cluster performance measures over other comparison methods (ARI, NMI, CA, FMI, SC) (including MultiK, SIMLR, scDeepCluster, SC3, scQcut, IKAP, CIDR, Seurat, SHARP, scGMAI, and DESC) with high scalability for large-scale datasets.	2022	[71]
Address the clustering of high missing rate or noisy scRNA-seq datasets	scCAEs	convolutional Auto-Encoders (CAE), Soft K-means clustering algorithm, KL divergence	Testing in eight real single-cell RNA-seq datasets covering different cell types, test results showed that scCAEs had higher ARI and NMI values than SIMLR, Seurat, SC3, SOUP and scziDesk in all datasets.	2022	[72]
Comparing the similarities and differences between positive and negative sample pairs, we learn more discriminatory feature representations and achieve more accurate clustering	scDCCA	AE, Contrastive learning, Deep embedded clustering, DEC	Fourteen real datasets reflect that scDCCA outperforms the eight state-of-the-art clustering methods in accuracy, generalization ability, scalability and efficiency, and scDCCA achieves the best or near best performance on CA, NMI and ARI	2023	[73]
Predicted for the expression of the target genes	DeepGRNCS	DNN	The model was validated using simulated datasets (based on Gaussian distribution, Boolean network and synthetic network) and real datasets (6 scRNA-seq datasets of BEELINE libraries and scRNA-seq datasets for lung cancer patients). The results indicate excellent AUROC and AUPRC values in all datasets.	2024	[74]
Functions as a prompting wrapper that efficiently annotates cell types by taking lists of marker or differentially expressed genes as input.	CelltypeGPT	GPT-4-API-based prompting wrapper (R package)	Using scRNA-seq data from different human and mouse tissues, GPTCelltype showed high agreement with higher average consistency scores than faster and lower cost of other automated annotation methods. It also showed good robustness and reproducibility in the simulation experiments	2024	[75]
Relies on GAT coupled with Network Enhancement for graph denoising to improve clustering performance.	scGAC	Graph Attention Networks (GAT), Network Enhancement, DEC-style self-training	Model clustering performance was evaluated using 16 real datasets, showing that the model achieved the highest ARI and NMI scores on multiple datasets, clustering results more similar to real labels, embedding cells in two-dimensional space using t-SNE or other dimension reduction methods, and clearer visualization results.	2022	[81]
Integrate information from attribute information AE and structural information AE based on attention mechanism to improve clustering performance	scDFC	Fusion Network	Eated using ten real single-cell RNA-sequencing datasets of varying sizes covering different species such as human, mouse and sponge, scDFC had the highest ARI and NMI scores, and the other four datasets also ranked the top three.	2023	[82]
Combines PageRank propagation with a ZINB Autoencoder and contrastive learning to map intricate cellular relationships.	scZAG	APPNP-style personalized PageRank propagation, ZINB Autoencoder, Graph contrastive learning Figure contrast learning, Self-optimized deep embedding clustering	Real scRNA-seq datasets from 10 different sequencing platforms were used to validate the performance of scZAG. The scZAG achieved the highest ARI scores on nine datasets and the highest NMI score on eight datasets. The Mann-Whitney U test results indicated significant differences between scZAG and other methods in ARI and NMI assessment indicators, further demonstrating the superior performance of scZAG.	2024	[83]
A hybrid deep framework integrating ELM mapping and triplet self-supervised learning to stabilize feature representation for clustering.	DCRELM	ELM-based random mapping, Dual-view perturbation, Autoencoder fusion, Triplet self-supervised learning	Using 12 real datasets with multiple cell types and states and six other state-of-the-art single cell clustering methods (scDeepCluster, GraphSCC, scGNN, DREAM, scDCCA, and scDFC), DCRELM outperformed the other methods in NMI, ARI, and F1 in most datasets.	2024	[84]
Utilizes a prototype-based deep neural network combined with classification-based testing to capture complex gene set distributions.	DeepGSEA	Prototype-based interpretable DNN, Classification-based significance testing	The ability of DeepGSEA was evaluated using four simulated scRNA-seq-seq datasets and three real scRNA-multiple phenoseq datasets. The results showed that DeepGSEA exhibited high sensitivity, interpretability and effective ability to process cellular heterogeneity.	2024	[91]
Converts gene-pair co-expression patterns into image-like histograms for supervised Gene Regulatory Network (GRN) inference.	DeepIMAGER	Convolutional Neural Networks (CNNs)	Compared with 10 existing Gene Regulatory Network inference methods (5 unsupervised and 5 supervised methods) on 6 different types of scRNA-seq and the corresponding ChIP-seq. DeepIMAGER achieves the highest AUROC score on all six datasets and has the minimum loss value and variance, outperforming other methods in prediction accuracy and robustness.	2024	[92]

**Table 3:** Application of partial machine learning in spatial transcriptomes.

Assignment	Model Name	Machine Learning Algorithm	Validation (Methods, Data Set, Results)	Published Time	Reference
To assess the degree of sample admixture in the potential space and to guide gene deletion value filling	gimVI	Variational Inference/Deep Generative Model	Validation was performed in 2 real mouse datasets and compared against scVI, Liger, Seurat and CORAL algorithms. The gimVI has the highest entropy mixing value for integrating cells into the joint latent space and the best k-NN purity on the mSMS dataset; for gene deletion filling, the Spearman correlation coefficient of gimVI is more than 38% higher than CORAL for the mSMS dataset.	2019	[99]
Integrating scRNA-seq and spatial transcriptome data to predict the expression patterns of unmeasured genes in the spatial transcriptome	SpaGE	PCA, SVD, KNN	The performance evaluation of SpaGE was conducted using six data sets (including one scRNA-seq data set from the same tissue and one spatial transcriptome data set). Compared with the three algorithms of Seurat, Liger, and gemVI, the correlation between SpaGE’s predicted value and the true value (such as Spearman correlation coefficient) was significantly higher than other algorithms, and the prediction performance of marker genes in different cell types was also better than other algorithms. Although SpaGE outperformed other algorithms in most cases, it is possible that because the KNN algorithm used by SpaGE is relatively simple, it may not be fully captured for some complex gene expression patterns	2020	[100]
Predict the expression of the unmeasured genes and effectively fill in the expression information of the measured genes	stPlus	AE, KNN	A large dataset, MERFISH_Moffit, containing 64,373 spatial transcriptomic cells and 31,299 scRNA-seq, was used to verify the model efficiency of stPlus. In comparison with the four baseline methods of SpaGE, Seurat, Liger and gemVI, the gene-level Spearman correlation coefficient, cell-level Spearman correlation coefficient, AMI, ARI, Homo and NMI were calculated for each dataset. The results showed that stPlus significantly outperformed the baseline method in all the metrics.	2021	[101]
The scRNA-seq data were spatially aligned with the other spatial data	Tangram	DL	Five snRNA-seq data from human and mice were used to evaluate the performance of Tangram in terms of cell type distribution consistency, gene expression prediction accuracy, low quality data correction, compared with STARmap, Visium, Allen brain map data and SHARE-seq, which showed that Tangram showed good performance on different datasets. Although the performance of Tangram is influenced by the dataset characteristics and the data quality, for most datasets, Tangram is able to generate reliable prediction results.	2021	[102]
Convolution of spatial transcriptomic data, cell type localization, cell-state analysis and analysis of tumor microenvironment	SPOTlight	non-negative matrix factorization (NMF), Non-negative Least Squares (NNLS)	Synthetic datasets and human pancreatic cancer, mouse scRNA-seq brain and ST real datasets were used to evaluate the performance of SPOTlight. Compared with other bulk data and convolution tools (MuSiC, CIBERSORTx, DeconRNA-seq, SCDC, RCTD, NMFreg) and CoGAPS, SPOTlight showed high prediction accuracy on shallow sequencing or small-scale scRNA-seq reference datasets, with JSD values (Jensen-Shannon divergence) close to 0, and the predicted cell type proportion highly similar to the true proportion.	2021	[103]
The graph structure information in the link graph is used to learn the composition of different cell types at each spatial point to improve the prediction accuracy of the model	DSTG	GCN	In two synthetic spatial datasets, the JSD value of DSTG was significantly lower than the SPOTlight method; in three real datasets, DSTG accurately predicted the cellular composition of each spatial point in the spatial transcriptome data and revealed the cellular spatial structure of tissues.	2021	[104]
The proportion of each cell type was inferred	SpatialDWLS	DWLS	A low-resolution simulated spatial transcriptome dataset, mouse brain spatial transcriptome dataset and human heart ST dataset were used for the validation of the model. Compared with other deconvolution methods (MuSiC, RCTD, SPOTlight and stereoscope) on the simulated data set, spatialDWLS performed well in both accuracy and computational efficiency. On real datasets, spatialDWLS can quickly analyze large spatial transcriptomic datasets and reveal dynamic changes in the spatial distribution of cell types.	2021	[104,105]
Decdown cell type mixtures and accurately predict the proportion of each cell type in each pixel	RCTD	MLE, LR	The comparison with unsupervised clustering, NMFreg, and DWLS, on 2 scRNA-seq datasets and three spatial transcriptomic data sets showed that RCTD can accurately distinguish cell types from unsupervised clustering and NMFreg, and can handle platform effects.	2022	[106]
Using features of spatial omics data to improve the accuracy of spatial domain identification and batch effect correction	DeepST	GNN	Eight datasets with different types and spatial resolution are used to verify the model, and compared with six spatial and identification algorithms. DeepST uses deep learning model for feature extraction and spatial information modeling, which can effectively process spatial omics data from different platforms and organizations and improve the accuracy of spatial domain identification.	2022	[107]
Classification cell types to generate more reliable single-cell spatial gene expression profiles	STCellbin	Leiden clustering, Bi-Directional ConvLSTM U-Net, Cellpose 2.0	Using two containing high quality images of nucleus and cell membrane/cell wall staining, and the corresponding spatial gene expression data set in comparing cell segmentation performance with three algorithms and compared with Baysor tool in terms of performance in downstream analysis, STCellbin outperforms other algorithms, especially in dividing cell membrane/cell wall.	2024	[108]

## Data Availability

No new data were created or analyzed in this study.

## Abbreviations

The following abbreviations are used in this manuscript:

NGS	Second-generation sequencing
ML	Machine Learning
SL	Supervised Learning
USL	Unsupervised Learning
DL	Deep Learning
SVM	Support Vector Machine
DT	Decision Tree
RF	Random Forest
t-SNE	T-distributed random neighborhood embedding
AE	Autoencoder
SAE	Stacked Autoencoder
RNN	Recurrent Neural Network
CNN	Convolutional Neural Networks
RNA-seq	RNA-Sequencing
RPKM	Reads Per Kilobase per Million mapped reads
FPKM	Fragments Per Kilobase per Million
TPM	Transcripts Per Million
KNN	K-Nearest Neighbor
RA	Rheumatoid arthritis
CART	Classification and regression tree
GBM	Gradient Boosting Machine
NNET	Neural Network
PLS	Partial Least Squares
scRNA-seq	single-cell RNA-sequencing
DAEs	Denoising Autoencoders
TF	Transcription Factor
GRN	Gene regulatory Network
GPT-4	Generative pre-trained transformer 4
GNN	Graph Neural Network
ELM	Extreme Learning Machines
CAE	Convolutional Autoencoders
VI	Variational Inference
GAN	Generative Adversarial Network
NMF	Non-negative matrix factorization
NNLS	Non-negative Least Squares
DSTG	Deconvoluting Spatial Transcriptomics Data Through Graph-based Artificial Intelligence
DWLS	Dampened Weighted Least Squares
RCTD	Robust Decomposition of Cell Type Mixtures
SAW	Spatial Analysis Workflow
